# Multi-Service Programs for Pregnant and Parenting Women with Substance Use Concerns: Women’s Perspectives on Why They Seek Help and Their Significant Changes

**DOI:** 10.3390/ijerph16183299

**Published:** 2019-09-08

**Authors:** Carol Hubberstey, Deborah Rutman, Rose A. Schmidt, Marilyn Van Bibber, Nancy Poole

**Affiliations:** 1 Principal, Nota Bene Consulting Group, Victoria, BC V8R 3M4, Canada (D.R.) (M.V.B.); 2 Centre of Excellence for Women’s Health, Vancouver, BC V6H 3N1, Canada (R.A.S.) (N.P.)

**Keywords:** gender, alcohol, substance use, FASD prevention, program evaluation, multi-service program delivery, client perspectives, pregnancy

## Abstract

Within Canada, several specialized multi-service prevention programs work with highly vulnerable pregnant and early parenting women with substance use issues. Experiences of trauma, mental health, poverty, and other factors associated with the social determinants of health complete the picture. Program evaluations have demonstrated their value, but less has been said as to women’s reasons for choosing to seek help from these programs, what they were hoping to gain, or what difference they believe has occurred as a result. The Co-creating Evidence project is a multi-year (2017–2020) national evaluation of holistic programs serving women at high risk of having an infant with prenatal alcohol or substance exposure. The evaluation uses a mixed methods design involving quarterly program output and “snapshot” client data, as well as in-person, semi-structured interviews and questionnaires with clients, program staff, and program partners. This article presents findings from interviews with women regarding why they sought help, how they used the services, and what they perceived to be the most significant change in their lives as a result. Obtaining help with substance use was the top theme for what women hoped to get from their participation in their program; however, women’s reasons were often intertwined. Additional motivations included wanting information, support or assistance with: child welfare; pregnancy; housing; getting connected to health care or prenatal care; and opportunities for peer support. With respect to the most significant life change, themes included: reduced substance use; improved housing; stronger mother–child connection; and improved wellness and social connections. Findings demonstrated that vulnerable, marginalized pregnant and parenting women who are using substances will seek help when health and social care services are configured in such a way as to take into consideration and address their unique roles, responsibilities, and realities.

## 1. Introduction

Prenatal consumption of alcohol and drugs continues to be a major health, social, and public policy issue in Canada [1,2]. Indeed, surveys have found that upward of 11% of women report consuming alcohol and between 1% and 5% reported using street drugs during pregnancy; both rates are considered to be underestimates given the inherent risks and stigma that go hand in hand with revealing prenatal consumption of alcohol and other substances. As well, a large percentage of the women who use substances prenatally are polysubstance users [3], with one report stating that the rate of (prenatal) poly substance “is as high as 50% in some studies” [4].

Women’s reasons for prenatal substance use are both complex and gendered. Research suggests that women’s prenatal substance use is often driven by a host of social determinants of health factors such as deep poverty, a history of physical or sexual abuse and neglect or other forms of trauma, intimate partner violence, mental health concerns, precarious living conditions including homelessness, child welfare involvement including maternal–child separation, and physical health problems [5,6,7,8]. Moreover, women who are struggling with substance use are typically isolated, are more likely to be living with a partner with problematic substance use, experience lower levels of social support, and have fewer resources at their disposal relative to their male counterparts [1,7,9]. These factors contribute to women’s reluctance to reveal the full extent of their substance use [3].

Systemic barriers compound the situation as standard systems of care often don’t meet the needs of women with prenatal or postnatal substance use issues, especially not women trying to raise children. Indeed, substance use and child protection services tend to operate in discrete silos with their own distinct goals, policies, expectations, and legislative responsibilities that in the past have resulted in high rates of child apprehensions from families wherein parental alcohol and drug use is a factor [10,11,12,13,14].

As a result, when seeking help for their substance use, vulnerable, marginalized women commonly experience numerous barriers including: stigmatization, lack of mental health supports, negative attitudes of health care providers, and adversarial approach of child welfare authorities [1,9,10,15]. Not surprisingly, fear of child welfare authorities is another factor in women’s avoidance of services, as is inadequate transportation and/or lack of child care [16]. These factors together make the decision to seek addiction treatment and support services by vulnerable, pregnant, and early parenting and substance using women all the more challenging. For service providers this also makes it all the more important that the programs they offer meet women’s needs [7,17,18,19].

Despite these hurdles, for many vulnerable women, pregnancy is a time of increased motivation to contemplate significant life changes, particularly prompted by women’s desire to keep their newborn in their care [20]. Indeed, the research literature suggests that women will respond to prevention services that are aimed at improving their health, including efforts to decrease or stop substance use or to increase their safer use of substances [19,20,21]. There also is strong evidence that outcomes for mothers and infants improve when accessible, women-centered substance use services or treatment are offered in conjunction with prenatal care [2,9,19,22]; moreover, care that is also tailored to the specific and evolving needs of women, their children, and the mother–child dyad is viewed as the most effective [12]. Programs that integrate practical and social supports with prenatal and postnatal health services, such as culture, transportation, child care, and meals, and that address the fear of child apprehensions may have an advantage in terms of engaging women who otherwise have few reasons to trust the formal health care system [23]. Moreover, programs that use non-judgmental, relationship-based, trauma-informed, and harm reduction approaches and that acknowledge women’s unique realities when it comes to the mother–child relationship have been found to be most effective in reaching vulnerable pregnant and parenting women with substance use issues [8,15,19,24,25].

Research indicates that many women who use substances during pregnancy are polysubstance-using [3,4]; further, once they engage with supportive services, they tend to be selective with respect to which substances they continue to use throughout pregnancy and which they reduce or stop using altogether [3]. In addition, women are inclined to underreport their substance use until they have built a relationship with their service provider that helps them to feel safe enough to disclose the full extent of their use [13]. Hence, community-based programs that have been leaders in the field of fetal alcohol spectrum disorder (FASD) prevention in Canada focus on problematic substance use more broadly, within the context of a social determinants of health context and women’s lived experiences as a way of engaging very vulnerable women without further stigmatizing them for their choices [22]. In keeping with this practice approach, in this study, “problematic substance use” is defined as the use of substances, including alcohol, that result in negative consequences in a person’s daily life, including adverse health consequences [26], as well as “social, financial, psychological, physical, or legal problems as a result of the drug use” [27].

Despite the evidence that exists with respect to promising approaches, further study would help to enhance our understanding of their implementation in community settings, including a better understanding of what motivates clients to seek services and supports as well as their perspectives on what changed the most in their lives as a result of their involvement in the service(s). To address this, a multi-site evaluation of “wrap-around” FASD prevention programs serving women at risk was envisaged.

### Co-Creating Evidence Evaluation Study

In keeping with the internationally recognized four-part FASD prevention model [28], women at highest risk of having an infant with prenatal alcohol exposure are those who who have substance use, mental health, and/or trauma-related issues and/or related social or financial concerns; “Level 3” FASD prevention programs offer holistic, multi-service programming to these women in ways that are specialized, culturally safe, and accessible. The Co-creating Evidence project, a multi-site three-year evaluation of eight different holistic wrap-around programs serving highly vulnerable women at high risk of having an infant with prenatal substance exposure and/or affected by FASD is the first of its kind in Canada. Funded by the Public Health Agency of Canada, the study runs from 2017 to 2020 and brings together many of Canada’s multi-service prevention programs with the aim of: sharing knowledge of their practices; demonstrating the effectiveness of prevention programming serving women with substance use and complex issues; and identifying characteristics that make these programs successful. The eight program sites volunteered to be part of the study.

Summary descriptions of the programs taking part in the study are provided in Box 1. The seventh program specifically serves pregnant or early parenting women who have substance use issues and/or other complex challenges. The eighth program serves women who are at risk by virtue of being young, i.e., 16 to 24 years of age; while problematic substance use may be an issue, it is not the program’s primary focus. Nevertheless, given the region’s very limited availability of substance use services, it is an issue that comes up with regularity. While the programs taking part in the co-creating evidence study are doing FASD prevention work, because they approach women’s issues holistically and employ a social determinants of health lens, the programs typically do not depict themselves as FASD prevention programs. At the same time, staff at all programs have training in FASD and trauma-informed practice (as well as other types of training) and also are members of a national FASD prevention research network. All of the programs have a mandate to support healthy birth outcomes, including helping to reduce the likelihood of FASD.

Box 1 **Capsule descriptions of co-creating evidence study’s program sites.**

**HerWay Home (Victoria BC)** offers drop-in and outreach support, on-site wellness and prenatal/post-natal groups as well as other health/medical services for women and their children, through a combination of program staff and in-kind support from the Island Health Authority. Women can participate in HWH until their child is approximately three years old.

**Sheway** (**Vancouver BC**) is a partnership between Vancouver Coastal Health Authority, Ministry for Children and Family Development, Vancouver Native Health Society and the YWCA of Vancouver. A range of on-site health and social services is offered on the first floor; an on-site health clinic is on the second floor; and child care and housing operated by the YWCA is on the third floor. Voluntary child welfare services are provided on-site through a partnership agreement with the provincial Ministry. The length of time that women can participate in Sheway is flexible and not set by the child’s age.

**Maxxine Wright (Surrey BC)** offers health and social supports through co-location with Atira Women’s Resource Society, which operates transition housing and second stage housing on-site. Atira offers most of the social programming, with participation from Fraser Health. Health/medical care is provided by Fraser Health. Child welfare and income assistance services are provided on-site through a partnership agreement with relevant provincial Ministries. Women can participate in MW until their child enters school.

**Healthy, Empowered, and Resilient (Edmonton AB)** is located within the Boyle Street Community Services, which provides an array of social, mental health, family, and cultural services in Edmonton’s downtown core. H.E.R. provides outreach to highly street-involved clients; through its staffing and partnership with Boyle McCauley Health Centre, H.E.R. clients have access to prenatal care and post-natal support. Women can participate in H.E.R. until six months post-partum.

**Raising Hope (Regina SK)** is a residential program located in an 18-unit apartment building (purchased by a non-profit housing society for the program’s exclusive use). A range of health/medical, social/cultural supports and programming including child care is offered on-site; residents are required to take part in daily programming. Women and their children can stay for 18 months.

**The Mothering Project (Winnipeg MB)** is a program of Mt Carmel Clinic and is co-located with the clinic. Through its staffing and partnerships, the MP offers a broad range of drop-in, outreach, and on-site supports and health/medical services along with a dedicated space for cultural ceremony. A licensed day care is co-located with MP with spaces set aside for program clients. Women can participate in the MP until their child reaches the age of five.

**Breaking the Cycle (Toronto ON)** is one of the first FASD prevention programs in Canada. The program provides children’s developmental assessment and mental health services with wrap-around services for women. Each woman is connected to a counsellor and each child is connected to a Child Development Worker. Women can participate in BTC until their child is six years old.

**Baby Basics (New Glasgow NS)** is a weekly drop-in parenting program operated by Kids First Family Resource Program, for women under age 25 and their children age 0–6. Although not specifically directed at women who are using substances, there are very few such options available to women in the region. BB offers a safe place for women to access support and talk about a range of issues. Women can participate in BB until their infant is one year old.

A previous article [29] described the study overall, with an emphasis on presenting: an overall theory of change for the programs; the services, activities and common components offered by the eight programs; women’s situations at intake; and interim qualitative findings regarding what clients like best about their program. This article shares additional interim findings from the study, based on data gathered between April and December 2018. In this article we focus on:

- What women hoped to get from engaging in their program;
- How women used their program’s services/activities; and
- The most significant changes that women experienced as a result of their program.

## 2. Materials and Methods

### 2.1. Study Design

The co-creating evidence study is employing a mixed-methods design, guided by principles of collaboration and partnership. The study is guided by collaborative and participatory principles [30], including the principles of “fostering meaningful relationships” (with program staff and stakeholders), “developing a shared understanding of the program,” “promoting appropriate participatory processes,” and “promoting evaluative thinking” [31,32]. In June 2017, the project team convened an introductory face-to-face meeting with program leaders to create a theory of change and the theoretical/philosophical foundations, approaches, activities, and anticipated outcomes of the programs collectively. Since that time, bi-monthly web-based teleconferences have been held to discuss key issues related to data collection and analysis, and to solicit the program managers’ feedback regarding interim project findings, draft reports, and knowledge translation. These meetings also provided the program leaders with opportunities to exchange information about their practices, shared issues, common understandings, programming shifts, and contextual issues of significance such as the ongoing opioid crisis. In addition, a National Advisory Committee was established at the beginning of the study, comprised of people with expertise in policy, programming, research and evaluation related to FASD. The Advisory Committee meets about 2–3 times a year.

### 2.2. Data Collection Processes and Instruments

Data are gathered through two separate processes:

1. The project team is undertaking two visits with each site, to conduct face-to-face, semi-structured, qualitative interviews, focus groups and questionnaires with staff, clients, and program partners.
2. The program manager/coordinator at each site is compiling and remitting quantitative output data and client-based data from April 2018 to September 2019, for a total of 18 months.

This article focuses on the qualitative interviews conducted with clients/participants during the first site visits between April and July 2018 and questionnaire data collected as part of the interview process. It is supplemented with interview data with staff and program managers/leaders.

### 2.3. On-site Data Collection by Project Team

The project team conducted individual qualitative interviews with clients, using an interview guide that was created for this project. Interview questions include: how the woman first learned about the program; what she hoped to get out of her involvement with the program; her life situation just prior to becoming involved with the program; her satisfaction with the program (e.g., what she liked most about the program, didn’t like, and would change); and what was most important to her about the program. As well, the interview contained open-ended questions pertaining to the client’s use of the program’s various services and activities. Clients were also asked about perceived impacts of the program. A modified version of the most significant change (MSC) technique was employed [33]; informants are asked to share what was “the most significant change that happened” as a result of the program. The MSC technique was modified in this study in that the analysis of clients’ stories did not involve formal review by external stakeholders or hierarchical selection and quantification of the stories.. The interviews were conducted in a private office, using a guided conversation approach that enabled interviewees to speak freely about which was most important to them.

After the interview, women were invited to complete the client questionnaire, which most often was administered verbally by the project team member, but which the participant could complete on her own if she preferred. The client questionnaire utilized a five-point Likert-type scale and focused both on participants’ experiences with their program (e.g., sense of physical safety and emotional safety; being respected; being a partner in planning and having a voice in decision-making; feeling that staff are sensitive when asking about difficult experiences, and so forth), and their perspectives on program impacts and how helpful the program had been in relation to outcomes in various facets of their life (e.g., in relation to accessing safe housing, accessing prenatal/postnatal care, and having a healthy birth, keeping or regaining their child(ren) in their care, and quitting or reducing their substance use). The questionnaire was created specifically for this study; at the same time, it included standardized questionnaire items that have been used in evaluations of trauma-informed and/or harm reduction focused programs [34].

All participants were provided with an honorarium ($25) for completing the interview and questionnaire. Program staff were available afterwards should clients have questions, comments, or concerns regarding the interview. Prior to launching the formal data collection, the project team pilot tested the interview guides and interview process with four sites. The purpose was to garner feedback from clients and staff regarding the process and the questions including how clients felt about answering potentially difficult questions about their lives.

### 2.4. Participants and Sampling Approach

Eligibility criteria for client participation in the interview and questionnaire were as follows: (a) the woman had to be accessing services from the program in the month of data collection; (b) women were 16 years or older; and, (c) women were English-speaking. Recruitment was handled by program staff who, approximately one month prior to the site visit, posted notices in program space regarding the interviews and made announcements during group and/or drop-in programming, inviting clients to come to the program for an interview or otherwise express their desire to do the interview off-site. Both forms of recruitment reinforced that the interviews were confidential and anonymous. For clients, a voluntary sampling approach was employed as program coordinators and the research team thought it was important for all clients who wanted to take part in an interview and who were available during the team’s site visit to be able to do so. At the same time, program staff did not formally track the number of clients whom they told about the site visit and interview opportunity, nor did they keep track of the number of clients who expressed disinterest in doing an interview; thus, it was not possible to know how many, if any, clients refused to participate in the in-person data collection process. A nominated sampling approach was used to create the sample of service partners; program staff at all sites provided the researchers with contact information for each program’s closest service partners.

A total of 125 program participants/clients (of whom 123 completed the client questionnaire) took part in an in-person interview between April and July 2018. The number of interviews with clients varied across sites, from *n* = 32 at one of the largest sites to *n* = 8 at two sites; there were three sites at which more than 20 clients were interviewed and five sites at which 8–11 women were interviewed. Differences in terms of the number of interviews conducted per site reflect the size and scale of the programs and was roughly proportional to the number of clients per site. As well, events outside of the program, including crises in the community and/or in clients’ lives also impacted response to the invitation to take part in the study.

All of the program participants identified their gender as female, and more than half were older than 30 years old (see Table 1). Every participant who completed the client questionnaire completed the demographic questions. Most often women self-identified their cultural background as Indigenous, followed by European/White. The length of time women had participated in the programs varied from less than one month to more than three years. Some of this variation is due to the policies of the individual program; for example, no participants from HER had participated in the program for more than one year as the discharge from the program occurs at six months post-partum.

### 2.5. Data Analysis

A frequency analysis of the client questionnaire was conducted in SPSS 26 (SPSS Inc., Chicago, IL, USA) to describe the participants. Missing data for each question ranged from *n* = 0 to *n* = 45, and percentages reported in the results did not include the women who did not answer each question in the denominator.

As the interviews with clients, staff and service partners involved open-ended questions, qualitative data analysis techniques were used, and qualitative data analysis software (NVivo12) (QRS International, Melbourne, Australia) was utilized to facilitate the analyses. In keeping with these techniques, written transcripts from all interviews were read multiple times by the researchers to begin the process of identifying themes and analytic ideas. Initially, each researcher coded the transcripts separately and identified preliminary themes inductively. The three researchers involved in carrying out the interviews highlighted naturally occurring patterns in the data, including words, phrases, or ideas most commonly voiced by participants, which formed the basis of the thematic analysis [35,36]. As means to strengthen the study’s rigor, the project team engaged in numerous discussions wherein they presented and reviewed one another’s emerging reflections, insights, and ideas about the data. Any differences in the researchers’ interpretations were resolved through discussion, review of the supportive textual evidence for each theme, knowledge of and comparisons with findings from the literature and previous relevant research, and consensus decision-making. Themes were ranked in strength based on a combination of the frequency with which they emerged and the intensity with which the speakers voiced the theme as evidenced by repetition of the theme/idea within the same utterance and/or the speaker’s emphasis or tone of voice when speaking, which was recorded by the researchers in their interview transcripts.

### 2.6. Ethics Approval

The evaluation study received ethics approval from the University of British Columbia Office of Research Ethics (H17-02168), Fraser Health Authority, Vancouver Coastal Health Authority, Island Health Authority, and York University. Study participants provided informed consent to participate in the interviews. All study participants were competent to provide their own informed consent and all were over age 18.

## 3. Results

### 3.1. Overview of the Programs’ Services

Drawing on interviews with staff and program leaders/managers and written program descriptions, Table 2 shows that, through a combination of their own staff or staff from partner organizations providing services on site, all co-creating evidence programs offer a mix of accessible health and social services and supports aimed at meeting clients’ holistic health, social, cultural, and practical needs. The programs’ core philosophical pillars include being relationship-based, trauma-informed, women-centered, culturally-informed, and employing non-stigmatizing harm reduction approaches. Many services were offered in group format, though nearly all programs offered one-to-one services as well. Programs connected to a health authority were more likely to offer health services on site (e.g., public health nurse, physician, nurse practitioner, or midwife).

### 3.2. What Women Hoped to Get from Participating in Their Program

Clients were asked what they had hoped to get by participating in their program. The themes were often interconnected, as clients generally provided several reasons for program involvement. The top themes to emerge were (in order of frequency):

- Wanted support in relation to problematic substance use and/or trauma
- Wanted support with child welfare and/or mother–child connection
- Wanted support and information related to pregnancy
- Wanted help in getting housing
- Wanted help in getting connected to health care or prenatal care
- Wanted healthy peer connections or peer support
- Brief discussion of the key themes follows.

#### 3.2.1. Wanted Support in Relation to Problematic Substance Use and/or Trauma

The most frequently emerging theme—voiced by nearly half of the clients interviewed—was that women were seeking help in addressing their substance use. However, for many women, this was intertwined with wanting help in dealing with effects of trauma because of experiences of violence or abuse and with wanting help with housing and with child welfare issues. (Child protection/child welfare terms are used interchangeably. Provinces/territories within Canada hold the legislated mandate to protect children and if need be to remove them from their parent(s). Generally speaking child protection social workers investigate and will remove children if deemed necessary, whereas child welfare social workers are tasked with working with the family to mitigate the risk factors. Nevertheless, any involvement of child protection/child welfare authorities means that the parent(s) is/are under scrutiny, that risk factors or concerns are present and that the parent(s) could lose their child if they are unable to satisfy the expectations of child welfare/child protection staff.)

I wanted to get sober. I wanted my children back, my family back. …. I was using drugs and alcohol. I was going through a rough time—breaking up with my partner who was abusive mentally and emotionally.

[I wanted] better housing, support to keep me away from drugs and alcohol, and help with nutrition. [I wanted] to keep my baby.

[I wanted] connections with other mothers and knowing that there were groups I could do that would help me with being a mother with trauma and addiction.

#### 3.2.2. Wanted Support with Child Welfare and/or Mother–Child Connection

Hand in hand with women’s desire for support in relation to their substance use was their desire for support in relation to keeping or regaining their child(ren) in their care and/or in having a strong mother–child connection. Clients often sought participation in specific program activities (e.g., substance use/recovery groups or parenting groups) and/or sought connection with program staff who would be able to speak to their motivation and capacity to care for their child(ren); most often, the programs were already known to clients for their support and advocacy in relation to child welfare.

I wanted a different way of bonding with my child, a different community.

I wanted connections with other mothers and knowing that there were groups I could do that would help me with being a mother with trauma and addiction.

I wanted sobriety and to learn to parent my kids; I had lost custody.

I wanted to get support and bring my child home and parent in a healthy lifestyle for her and for me.

While wanting to demonstrate that they were capable of parenting may have been the initial impetus for attending their program, as women made gains, some realized that they wanted more, whether that was a better quality of life or a more stable foundation for building a life for their children.

To start with, I only wanted to get my children back. Now I want a better quality of life for me and my kids.

I also wanted to connect with a therapist. I wanted to have a stable foundation to work with. I knew [child protection] would be involved, so I wanted to create a healthy foundation for that involvement.

#### 3.2.3. Wanted Support and Information Related to Pregnancy

Another strong theme and reason for engagement with their program was a desire for guidance and information in relation to their pregnancy as well as help with other issues such as finding safe and stable housing, child welfare, and substance use. Many women spoke of wanting this pregnancy to be different from their previous one(s).

I was pregnant and had addictions and I wanted to have support through my pregnancy.

Initially, I didn’t know where or how I’d go with the pregnancy. I needed guidance and support. When I first found out I was pregnant, I cried for 48 hours straight. I also wanted information. I didn’t know where to go and what my next step would be.

Support to keep moving forward. I wanted to make changes in my life.

#### 3.2.4. Wanted Help in Getting Housing

As noted previously, at intake over half of clients (ranging up to 85% at some program sites) had precarious and/or inadequate housing. Clients voiced their desire for program assistance in helping them access safe and stable housing, as they also recognized the inextricable connection between housing and child welfare authorities’ safety concerns.

Support, because I have [child protection] involvement. I wanted support and stable housing and getting addictions out of the way. I want to go home with my baby.

I was in a really shitty situation. I was living with a friend; she was using drugs. I ended up in a shelter. I needed resources to help me with my pregnancy and with raising my baby, and I wanted help getting into different housing.

#### 3.2.5. Wanted Help in Getting Connected to Health Care or Prenatal Care

Approximately 20% of the clients interviewed stated that they were looking for health or prenatal care. Some women sought a health/prenatal care provider because they had recently moved to the community—often to flee an abusive ex-partner or to shed ties with people who used substances—while others may have been in the community for a while but sought a prenatal care provider in order to focus on having a healthy pregnancy. As well, some women emphasized that they wanted to connect with a health or perinatal care provider who would not judge or stigmatize them for their substance use.

My family doctor set me up with a maternity doctor who specialized in working with women with addictions. She suggested that I network with someone. I was afraid that the nurses at the hospital would see my medical history, see that I was on suboxone, and be judgmental and call child welfare.

I was looking for prenatal care. I was looking for programs and people to help support me to have a healthy pregnancy.

#### 3.2.6. Wanted Healthy Peer Connections or Peer Support

Finally, wrapped up in the notion of finding support was a desire by clients for healthy peer connections, partly born of a desire to be amongst women with similar backgrounds and with whom they could safely share their story.

I was looking for support and advocacy for my situation. I was needing a group for women like me who have been through years of trauma and abuse. I did not want to be the only woman in the room with that kind of lived experience.

[I wanted] to open up more; learn how to speak to others when they needed help; to share my story with others who were struggling; to help guide others on a positive path.

Some women wanted the sense of community that could come from being in a prenatal group and to have answers to their questions.

The sense of community here. Support group. I didn’t know other pregnant women.

### 3.3. Clients’ Experiences of Utilizing Their Program’s Services/Activities

As part of the qualitative interviews, clients were asked to describe ways in which they used the various activities or services offered by programs. A sample of their comments, presented in Table 3, provides a more complete picture of the services listed in Table 2 and sheds light on women’s perspectives on the value of a “one-stop” approach to health care, poverty, child protection issues, parenting, social connections, substance use, and culture.

### 3.4. Most Significant Change(s) That Women Experienced (as a Result of Their Program)

As a key component of the qualitative interview, clients were asked what had been the most significant change(s) that had taken place for them and their family since they started participating in their program. As was the case with other open-ended interview questions, women’s responses often contained multiple themes, and the themes were clearly intertwined. The top themes were (reported in order of frequency):

- Quit or reduced substance use;
- Strengthened mother–child connection;
- Kept/regained custody/care of child(ren);
- Improved wellness/mental health;
- Increased support;
- Safer, improved housing.

#### 3.4.1. Quit or Reduced Substance Use

The most frequently emerging theme of the “most significant change,” voiced by approximately 40% of the clients interviewed (*n* = 51), was that they had quit or reduced their substance use. Many attributed their program with helping them to quit using substances.

If I hadn’t been at this program, it would have been harder to stay sober, and my baby would have gone to live with my mom.

Because the staff care so much about their clients, I’ve gotten clean. I’ve been in and out of addiction for 18 years, but because of them, my using time has reduced—it’s down to two days. They’ve reached out to me. They’ve made a huge difference to me.

In keeping with these findings, on the client questionnaire, 79% (*n* = 93, 4% missing;) agreed or strongly agreed that the program had helped them quit, reduce or engage in safer substance use, and 70% (*n* = 83, 3% missing) agreed or strongly agreed that the program had helped them access substance use services or supports. Approximately 20% of women indicated on the client questionnaire that these items were not applicable to them, as they had not sought help from their program for substance use concerns.

#### 3.4.2. Strengthened Mother–Child Connection

The second theme, voiced by nearly the same number of clients (*n* = 49), pertained to the existence or strengthening of the connection between the woman and her child(ren). This important theme focused on the presence and preservation of the mother–child relationship rather than on whether women had retained or regained custody of their child(ren), and thus it was voiced both by women who did not have their child(ren) in their care as well as those who did. Similarly, most women (*n* = 93, 79%, 5% missing) agreed or strongly agreed on the questionnaire that the program had helped them improve their connection to their children. This theme also relates to the situation voiced by some women that, had they not had support from their program, they likely would not have continued with their pregnancy:

My baby and I have a home. We know we’re not alone—both because I can call the [program] staff and because of other women. Without [program], I probably wouldn’t have had my baby.

My stress level has gone down quite a bit. I know that no matter what, they’ll be here. The program helps with everything: prenatal care, housing, [child protection] advocacy, baby stuff. Before coming into this program, I felt hopeless.

#### 3.4.3. Kept/Regained Custody/Care of Child(ren)

In keeping with the previous point, the third strongest theme was that women had retained and/or regained their children in their care. As reflected in clients’ comments, keeping their infant in their care and/or getting their child back from foster care nearly always occurred in tandem with other pivotal life events, such as reducing or ending their substance use, accessing stable housing, and breaking away from a high-risk “past lifestyle.”

Getting my daughter back from foster care and having my baby come home from the hospital with me. Getting my kids back is the biggest thing. That showed me I’m done with my past lifestyle.

I’ve been clean and sober for 22 months. Getting clean changed my whole life. I got my son back, and I’m about to get the older two children back in September.

#### 3.4.4. Improved Wellness/Mental Health

The fourth top theme, voiced by about a third of the clients interviewed (*n* = 39), had to do with women’s experience of improved wellness and well-being. Along these lines, clients described feeling happier, less stressed, more self-confident, self-aware and emotionally equipped to deal with personal triggers, as well as being more socially engaged.

Our household is more balanced. I know my triggers and deal with anger better. I am more balanced emotionally.

Coming to [the program] is getting us out of our shells. My daughter and I, we really needed this. It’s really made a difference in terms of our health, mental health and well-being

#### 3.4.5. Increased Support

Strongly connected to all themes was clients’ sense that they had increased supports—both from program staff and from other program participants—and a support network that they could count on. As well, for some women, a significant change was their newfound capacity to reach out to others for support when needed.

I’m about to reach out for support. I couldn’t do that before.

Paralleling these findings, on the client questionnaire, 93% (*n* = 111, 3% missing) agreed or strongly agreed with the statement “I feel supported and less isolated; I have social support.”

#### 3.4.6. Safer, Improved Housing

Approximately 25% of the clients interviewed reported that, for them, a/the significant change since becoming involved with their program was accessing safe and adequate housing. As noted previously, clients often spoke of housing in essentially the same breath as they talked about keeping/regaining their child(ren) and/or quitting or reducing their substance use, as the inter-connections between these outcomes were evident. In one client’s words:

Getting suitable housing and reuniting with my son. We were in the single room occupancy apartment when I had the baby. Then he went into a foster home. Then we got housing and the baby was returned to us.

#### 3.4.7. Additional “Significant Changes”/Outcomes

Three additional “most significant change” themes are important to mention, given that they emerged in the comments of quite a few clients (*n* = 20 or roughly 16% of those interviewed). These were:

- Increased self-confidence/self-esteem;
- Reduced isolation and/or increased connection to peers;
- Increased self-compassion/self-determination.

As these clients stated:

They’ve helped me open up more. I feel more self-confident and happier. I’ve opened up a lot more.

I got back into my culture. I’m teaching my daughter how to smudge and do drumming.

I’m happy, have lots of friends. I’ve connected again with family, and I’m sober.

It helped me—the therapy and the groups—to reflect on myself, and I wanted to do that for myself. I have a better understanding of myself. They give us the tools to help ourselves.

### 3.5. Summary of Key Findings

By way of summary, Table 4 presents the top themes and their interconnection in relation to what women hoped to get from participating in their program and their most significant change.

## 4. Discussion

The interim findings of the Co-creating Evidence multi-site evaluation make a valuable contribution to the literature by focusing on the perspectives of highly vulnerable, pregnant, and early parenting women with problematic substance use, and drawing an arc from their life circumstances prior to entering the program, to what it was they were hoping to gain by reaching out for help, and finally, to their views on the most significant changes in their lives as a result of their involvement with the program. In doing so, the preliminary findings support the already rich literature on the complexity of issues that this population of women face, including intimate partner violence, trauma/mental health, poverty, precarious housing, and child welfare involvement [1,5,6].

The study also contributes to a better understanding as to what prompts women to want to make a change in their life circumstances. In this vein, the study affirms the view that pregnancy can be a powerful catalyst for transformation for women who are marginalized from mainstream services by virtue of their circumstances and contributes to the literature that vulnerable pregnant and parenting women experiencing numerous personal and systemic barriers will seek help, ideally at a single point of access, when those services are non-judgmental and take into consideration and address their realities [2,19,37,38,39]. Additionally, from a gender perspective, women have also been found to respond to and benefit from programs that take into consideration their unique roles and responsibilities and that reduce obstacles to their participation, including those related to caring for children and family [18,19,40].

On that note, women’s top priorities prior to joining their program and the areas of their lives in which they reported significant improvement were intertwined such that they rarely spoke of just one priority or benefit. In this regard, while obtaining support in relation to their problematic substance use was the most frequently cited theme, it was often entwined with issues of current or past trauma. Addressing these issues together was part of the programs’ holistic, trauma-informed, and women-centered approach. Often closely associated with clients’ desire for support in relation to their substance use was a yearning to retain and/or regain their children; a related goal was to create a better life for themselves and for their child(ren) including learning more about parenting and having opportunities for positive peer connections for themselves and their child(ren). This lends further weight to the tenet that when working with vulnerable women, the child is “undeniably part of the equation” [40]. At the same time, women who use substances pre and postnatally often experience parenting difficulties resulting in the further likelihood of child welfare involvement; hence, recommended best practices include not only being responsive to the mother–child dyad but also development of a collaborative working relationship between substance use services and child welfare agencies [12,23,41].

For almost one-quarter of clients, improved housing was reported as a, if not the, most significant change in their lives. As four of the co-creating evidence study programs offered housing to at least some clients either on-site (for example, through the program’s own services or through co-location with a housing agency) and others achieved this through partnerships with local or provincial housing providers, clients were able to more readily access supported/social housing as a result of their association with their program. Safe and stable housing is fundamental to satisfying the safety concerns of child welfare authorities—i.e., enabling women to be able to go home with their infant after giving birth and/or to regain custody of older children—as well as to sustaining other positive life changes.

Poor health or mental health were key issues at intake and were areas in which clients experienced positive change as a result of their involvement with the programs. For women, this “significant change” was characterized as an overall sense of wellness and social connections; a frequent theme was their experience of improved mental well-being, increased support, and self-confidence/self-esteem, reduced isolation, and increased self-compassion and self-determination. In describing the pathways that mothering women take toward quitting their substance use, Marcellus similarly found that restoring their sense of self—described as gaining and sustaining recovery, becoming more socially connected and less isolated, improving personal well-being, and regaining credibility in multiple domains—was a key trajectory for women [42]. In this regard the findings to date of the co-creating evidence study are consistent with client perceptions of integrated treatment programs in Ontario, in that those participating in integrated programs reported positive psycho-social outcomes, including improved self-confidence and greater sense of self [2].

Finally, it has also been noted in the literature that women respond differently to substance use services than do men, showing a preference for services and programs that engender an atmosphere of hope, acceptance, and support [43]. Earlier qualitative findings from the Co-creating Evidence study [29] described that what clients liked best about their program was the caring, non-judgmental, supportive, helpful approach of staff. This along with the availability of multiple services in one place was among the top themes, indicating that it is possible for vulnerable pregnant and parenting women with complex challenges including problematic substance use to achieve positive outcomes when presented with the right mix of services and approaches that adequately address their health and social support needs as women and mothers.

### Limitations

Despite the strong congruence between this study’s findings and the existing literature, the study’s limitations should be noted. With regard to the on-site client-related data collection (i.e., interviews and questionnaires with clients), we understand that the voluntary sampling approach could have resulted in biases, in that clients with more positive views about their program would have been disproportionately inclined to take part in the evaluation study. As well, without the denominator in terms of potential participation in the evaluation, we cannot assess bias nor the representativeness of the sample. Further, with a circumscribed number of days for each site visit and on-site data collection, clients had a narrow window of opportunity to take part. As such, we cannot know for certain that we achieved “saturation,” nor was the concept of saturation the means by which we determined the number of interviews to conduct at each site. Nonetheless, we have no reason to believe that clients who held fewer positive perspectives were disinclined to participate in the study nor were they prevented from doing so. The confidential, conversational approach to interviewing also facilitated participants sharing their diverse experiences and perspectives. Given that there will be a second round of on-site data collection with clients, there will be an opportunity to explore the issue of sampling bias and determine whether saturation was achieved.

## 5. Conclusions

Women’s prenatal alcohol use and other substance use frequently occur within the context of inadequate housing, intimate partner violence, trauma, poverty, and social isolation. These burdens combined with systemic barriers affect their ability and willingness to engage with formal health care services. Often, women reveal the full extent of their substance use, including alcohol, only when they feel safe, accepted, not stigmatized, and when their program/service is meeting their practical needs.

With its focus on clients’ perspectives, this paper makes a valuable contribution to the literature regarding multi-service programs aimed at vulnerable pregnant and parenting women who use alcohol and other substances. The article highlights the multiple, interconnected reasons why women seek help from these programs, most notably the twin desires to address their substance use and to regain and/or keep their baby/children in their care. Rounding this out are women’s desires for help with housing, prenatal/health care, and peer support, suggesting that pregnancy can be an important catalyst for making significant life changes.

This study affirms how capably women can provide such guidance to service providers as to their service needs. It also affirms the value of a holistic approach that addresses both problematic substance use and the social determinants of health in accessible, women-centered, and integrated programming.

To ensure that this population of vulnerable pregnant/parenting women receive such holistic services, funders too will need to consider how to integrate funding streams, to include health, social and cultural services, housing, income support, child welfare, and public safety resources to these multi-service programs.

## Figures and Tables

**Table 1:** Characteristics of client questionnaire participants.

Program Site	Herway Home	Sheway	Maxx Wright	HER	Raising Hope	Mothering Project	Breaking the Cycle	Baby Basics	Total
	*n*	*n*	*n*	*n*	*n*	*n*	*n*	*n*	*N* (%)
**Number of participants**									
	8	35	20	9	10	25	8	8	123
**Age**									
16–24	1	4	3	5	3	3	1	6	26 (21%)
25–30	2	5	6	3	5	5	3	2	31 (25%)
30+	5	26	11	1	2	17	4	0	66 (54%)
**Cultural background (top 3)**									
Indigenous	2	19	8	7	8	20	0	1	65 (53%)
European/White	3	9	4	1	2	2	5	7	33 (27%)
Mixed Race	3	6	5	0	0	3	1	0	18 (15%)
**Length of time participating in program**									
<1 month	0	0	1	1	0	0	0	1	3 (2%)
1–6 months	3	3	3	4	5	5	3	1	27 (22%)
7–12 months	1	5	2	4	3	2	2	1	20 (16%)
1–3 years	3	12	4	0	1	7	3	3	33 (27%)
>3 years	1	15	10	0	1	11	0	2	40 (33%)

**Table 2:** Services/activities offered by programs via staff or service partners or via referrals.

Service/Activity	Number of Programs Offering Service/Activity on Site via Program Staff or Service Partners	Number of Programs Linking Clients to Service/Activity via Referral to Service Partners	Total Number of Programs Offering or Facilitating Access to Service
Basic needs support	8	0	8
Child assessment and early intervention	5	2	7
Child care on site	7	0	7
Child health	6	2	8
Child welfare support	7	0	7
Cultural programming	5	1	6
Drop in; peer connection	8	0	8
Food; nutrition	8	0	8
Health; medical services	6	2	8
Housing	4	4	8
Life skills	6	1	7
Mental health; trauma	8	0	8
Outreach	6	0	6
Parenting programs	7	1	8
Prenatal and postnatal care	7	1	8
Substance use counselling	7	1	8

**Table 3:** Examples of ways that clients utilized their program’s services.

Service/Activity	Examples of Ways That Clients Utilized the Service/Activity
Basic needs support	Lots of practical support such as a bag of clothes for the baby and items for myself such as a sports bra when I gave birth recently. I got help with income security.
Child assessment and early intervention	A speech language pathologist was linked to my son—the referral from [the program] sped up the process. We were referred to the hospital and introduced to a dentist for the children. The staff made sure the children were up to date for their immunizations and referred them to an Infant Development worker.
Child care on site	My baby is in daycare sometimes. I want to get a regular spot in the daycare. They have the daycare here. You can take “self-care” breaks and have the kids go into daycare.
Child health	My son had vaccinations with the Public Health Nurse and saw the dental hygienist. He is getting dental surgery soon as a result. I brought my older daughter to see the doctor; my baby is in care still.
Child welfare support	When my partner assaulted me in November, child welfare was automatically involved. I was concerned they would take my kids away. I met with child protection services with [program] as my support. [The program] advocated for me to get my kids back early and to have visits, and then extended visits with my kids—and then my kids came back to me.
Cultural programming	I went to the Round Dance organized by [program’s partner organization]. I do drumming and Talking Circle here.
Drop in; peer connection	A lot of the women who come here I’ve known since childhood. Now we’re moms together. All my friends are here; they’re getting sober and are moms. The other women are role models—they help us develop skills and increase confidence. I have connections with other women coming to [the program].
Food; nutrition	Lunch program; weekly bag of nutrition; fresh bread; prenatal and postnatal minerals and supplements plus education and workshops on healthy nutrition. Every week I get a four-liter jug of milk, eggs and cheese. That weekly food bag really helps.
Health; medical	I see the Public Health Nurse at the [program] regularly. She is the one who wanted me on Methadone—she said would it be better for the baby than T3s. We discuss sexual health information in group.
Housing	I got help with housing through another agency but [the program] helped me to switch the lease to be in my name, which means that I have to be more responsible. My support worker got me into a nice place for women. People are getting their kids back and staying off drugs.
Life skills	We had someone come and talk about the food guide. The nurse from [program’s partner organization] provided nutrition education. We learned how to make baby food and we did fire prevention education.
Mental health; trauma	I spoke with the trauma counsellor about some of the things I have seen in the last year. I’ve been living a very high-risk life—drugs, violence, lots of money. I’m working with the trauma worker now. I’m doing that to get my son back.
Outreach	My Outreach Worker is my mainstay. My Outreach Worker will go to court with me as an advocate.
Parenting programs	I took a couple of parenting groups. That helps me with how I play with my daughter. I learned about her development and how to talk with my teenage son. I’ve done lots of programs, like wellness, circle of security, and rediscovering parenting. They are all very useful because as my kids age, new problems arise and I retake the group to see how the information applies to my current situation. I like that the groups repeat regularly.
Prenatal; postnatal care	I’m seeing the doctor here, getting Methadone, and going to prenatal classes. They give me rides to [program’s partner organization] for prenatal care. We learn about breastfeeding, traditional teachings, abuse and violence there.
Substance use counselling	I had stopped cocaine and alcohol in 2017. [The program] helps me deal with and address the urges. Fear of [child protection] also motivates me. I have a one-to-one counsellor I was seeing weekly until recently. Now we meet biweekly. I’m going to be starting Group (struggling with addictions group) tomorrow. I talk with the counsellor all the time about my use and where I live. It is hard to stop using when you are selling drugs all the time.

**Table 4:** Top themes in relation to what women hoped to get from participating in their program and their most significant change.

What Women Hoped to Get from Participating in Their Program	Women’s Most Significant Change
Support with problematic substance use and/or trauma (*n* = 61)	Quit, reduced or safer substance use (*n* = 51) Improved wellness/mental health (*n* = 39)
Support with child welfare and/or mother-child connection (*n* = 58)	Strengthened mother – child connection (*n* = 49) Women keep/regain children in their care (*n* = 43)
Support and information re: pregnancy (*n* = 37) Help in accessing health care or prenatal care (*n* = 29)	Increased support (*n* = 34)
Help in getting safe, stable housing (*n* = 32)	Safer, improved housing (*n* = 29)
Healthy peer connections or peer support (*n* = 23)	Reduced isolation/connection to identity, peers, culture (*n* = 19)
